# Retraction: A nomogram for predicting metabolic-associated fatty liver disease in non-obese newly diagnosed type 2 diabetes patients

**DOI:** 10.3389/fendo.2026.1938497

**Published:** 2026-07-20

**Authors:** Frontiers Editorial Office

The authors retract the article cited above.

Following publication, the authors contacted the Editorial Office to request the retraction of the cited article, stating that the sensitivity and specificity values presented in the ROC curve analysis are incorrect. These inaccuracies have compromised the reliability and validity of the study results. The conclusions reported in the article are no longer supported by the analyses.

This retraction was approved by the Chief Editors of Frontiers in Endocrinology and the Chief Executive Editor of Frontiers.

The authors present updated findings in a new manuscript: Cheng Y, Li F, Cui Y, Li T, Sun W and Shi H (2026). A nomogram for predicting metabolic-associated fatty liver disease in non-obese patients with newly diagnosed type 2 diabetes. *Front. Endocrinol*. 17:1846441. doi: 10.3389/fendo.2026.1846441.

